# Cáncer de mama en seis familias del Tolima y el Huila: mutación *BRCA1* 3450del4

**DOI:** 10.7705/biomedica.4673

**Published:** 2020-03-30

**Authors:** Jennyfer Benavides, John Suárez, Ana Estrada, Mábel Bohórquez, Carolina Ramírez, Justo Olaya, Yesid Sánchez, Gilbert Mateus, Luis Carvajal, María Magdalena Echeverry

**Affiliations:** 1 Grupo de Citogenética, Filogenia y Evolución de Poblaciones, Universidad del Tolima, Ibagué, Colombia Universidad del Tolima Universidad del Tolima Ibagué Colombia; 2 Unidad de Oncología, Hospital Universitario Hernando Moncaleano Perdomo, Neiva, Colombia Hospital Universitario Hernando Moncaleano Perdomo Neiva Colombia; 3 Programa de Medicina, Universidad del Tolima, Ibagué, Colombia Universidad del Tolima Universidad del Tolima Ibagué Colombia; 4 Unidad de Oncología, Hospital Federico Lleras Acosta, Ibagué, Colombia Hospital Federico Lleras Acosta Ibagué Colombia; 5 Genome Center and Department of Biochemistry and Molecular Medicine, University of California, Davis, USA; Unidad de Oncología, Fundación Genética y Genómica, Medellín, Colombia University of California University of California Davis USA

**Keywords:** neoplasias de la mama, genética, genes *BRCA1,* mutación, deleción cromosómica, Breast neoplasms/genetics, genes, *BRCA1*, mutation, chromosome deletion

## Abstract

**Introducción.:**

El cáncer de mama es un problema mundial de salud pública; entre el 5 y el 10 % de los casos presentan agregación familiar, lo que se explicaría por la presencia de mutaciones en genes de alto riesgo como el *BRCA1* y el *BRCA2.* El origen fundador de la deleción *BRCA1* 3450del4 en Colombia ya fue reportado.

**Objetivo.:**

Hacer un análisis descriptivo de seis familias del del Tolima y del Huila con la deleción *BRCA1* 3450del4 de la asociación de la mutación germinal, con el cáncer de mama y la agregación familiar.

**Materiales y métodos.:**

Se hizo un estudio descriptivo y transversal de seis casos índice con cáncer de mama positivos para *BRCA1* 3450del4, que cumplían tres de los criterios establecidos por Jalkh, *et al.* A partir de la información de las entrevistas, se realizaron los árboles genealógicos (GenoPro™, versión 2016). Se tipificó la mutación en familiares sanos y afectados que aceptaron participar.

**Resultados.:**

De los 78 individuos seleccionados por conveniencia en las seis familias, 30 presentaron la mutación *BRCA1* 3450del4; de ellos, seis tenían cáncer de mama, uno, cáncer de ovario, uno, cáncer de mama y ovario, y otro, cáncer de próstata; 21 no presentaban neoplasias. De los 30 individuos portadores de la variante patogénica, seis eran hombres y 24 mujeres, 13 de ellas menores de 30 años.

**Conclusiones.:**

En este estudio se confirmó la asociación de la deleción *BRCA1* 3450del4 con el cáncer de mama de agregación familiar.

## Abstract (en)

**Introduction:**

Breast cancer is a worldwide public health problem; between 5% and 10% of the cases present familial aggregation explained by genes of high risk such as *BRCA1* and *BRCA2.* The founding origin of the deletion *BRCA1* 3450del4 in Colombia has been previously reported.

**Objective:**

To carry out in six families from Tolima and Huila departments a descriptive analysis of the presence of the *BRCA1* 3450del4 mutation associated with breast cancer and familial aggregation.

**Materials and methods:**

We conducted a descriptive and cross-sectional study of six index cases with breast cancer positive for *BRCA1* 3450del4 that fulfilled three of the criteria established by Jalkh, *et al.* The genealogical trees were made using the information of the interview data (GenoPro™, version 2016). The mutation was typified in healthy and affected relatives who agreed to participate.

**Results:**

Thirty of the 78 individuals selected by convenience in the six families presented the mutation *BRCA1* 3450del4 six of whom developed breast cancer, one, ovarian cancer, one ovarian and breast cancer, and one prostate cancer; 21 did not present any type of neoplasm at the time of the study. Of the 30 individuals carrying the pathogenic variant, six were men, 24 were women, and 13 of these were under 30.

**Conclusions:**

In this study of families with the deletion *BRCA1* 3450del4 in Tolima and Huila we confirmed its association with familial aggregation of breast cancer.

El cáncer de mama se considera un problema de salud pública a nivel mundial. Es la primera causa de morbilidad y la segunda de muerte, por cáncer en mujeres. En Colombia, es la principal causa de morbimortalidad por cáncer en mujeres, con una tasa de incidencia del 44,1 % y una mortalidad del 11,9 % ^1^.

Todos los tipos de cáncer presentan un componente genético que incide en la predisposición a desarrollar la enfermedad. Este puede ser de origen germinal, que genera la agregación familiar, o esporádico, de origen somático y sin agregación familiar ^2^. En el cáncer de mama, la agregación familiar, explicada por la presencia de variantes patogénicas de origen germinal de alto riesgo, ocurre aproximadamente en 5 a 10 % de los casos, en tanto que en el 90 % restante se considera de origen esporádico ^2^^-^^4^.

La mayor parte de las variantes germinales de riesgo corresponde a genes supresores de tumores, entre los que se cuentan el *BRCA 1* y el *BRCA* 2, cuyas variantes, consideradas de alto riesgo, se han asociado con una historia familiar que responde al patrón de herencia autosómica dominante ^2^^,^^5^^,^^6^. Se estima que en las mujeres portadoras de variantes en el *BRCA1* y el *BRCA2,* el riesgo de desarrollar cáncer de mama se incrementa en 87 % y, el de cáncer de ovario, en un 68 % ^7^^-^^9^. Además, se considera que las mutaciones en el *BRCA* explican entre el 16 y el 40 % de este tipo de cáncer con agregación familiar ^10^^,^^11^.

En el gen *BRCA1* del cromosoma 17q21, se han descrito 600 variantes alélicas, aproximadamente, entre ellas, la patogénica 3450del4, ubicada en el exón 11, como resultado de la deleción del codón Gln 1111 (CAA) y el primer nucleótido (G) del codón 1112, lo que produce un codón de terminación anticipada en la posición 1115 ^12^. Esta es una de las mutaciones fundadoras de riesgo presentes en el país: en 12 de los 766 casos de cáncer de mama estudiados por Torres, *e tal.,* en el 2009 ^13^ y en 11,5 % de los casos de cáncer de ovario estudiados por Rodríguez, *et al.*^14^, en tanto que en el artículo de revisión de Ossa, *et al.,* se reporta en Brasil y Chile ^15^.

En este estudio, se hizo un análisis descriptivo para determinar la asociación de la mutación *BRCA1* 3450del4 con la presencia de cáncer germinal en seis familias de los departamentos del Tolima y Huila.

## Materiales y métodos

### Antecedentes metodológicos

Los casos índice se tomaron de los proyectos *Genetic analyses of breast cancer in admixed populations* y de una tesis de maestría ^16^, trabajos desarrollados por el Grupo de Investigación Citogenética, Filogenia y Evolución de Poblaciones (GCFEP) de la Universidad del Tolima, en asociación con el Instituto Nacional de Cancerología, la Universidad de California-Davis y la Universidad de Oxford, y financiados por Glaxo Smith Kline Oncology (GSK), parte de cuyos datos genotípicos fueron publicados en el 2014 en otro artículo ^17^.

### Muestra

Se seleccionaron seis casos índice de los 28 con agregación familiar provenientes del del Tolima y el Huila que presentaron la mutación *BRCA1* 3450del4, siguiendo tres de los criterios de inclusión propuestos por Jalkh, *et al.*^10^, en el 2017: 1) pacientes con cáncer de mama de menos de 40 años de edad en el momento del diagnóstico; 2) dos o más integrantes de la familia afectados con cáncer, y 3) por lo menos, un familiar afectado con este tipo de cáncer.

### Toma de muestras

Se tomaron dos muestras de sangre periférica de 4 ml a los familiares del caso índice que se pudieron contactar y aceptaron participar.

### Construcción de árboles genealógicos

La ampliación de los árboles genealógicos de los seis casos índice se basó en: a) la entrevista del caso índice; b) las visitas a las familias; c) la revisión de registros eclesiásticos; d) las entrevistas a diferentes parientes, y e) el uso del programa GenoPro™, versión 2016.

### Extracción de ADN y genotipificación de la mutación

Se aisló el ADN de cada uno de los miembros muestreados en las seis familias portadoras de la mutación *BRCA1* 3450del4 a partir de 430 µl de sangre total, empleando el equipo automatizado Maxwell™ y el estuche de extracción Maxwell 16 Blood DNA Purification Kit™ (Promega). La cuantificación se hizo con el espectrofotómetro Nanodrop ND-2000™. Para la genotipificación, se utilizó una reacción en cadena de la polimerasa (PCR) competitiva específica para alelo con el sistema de genotipificación KASP™ (LGC Genomics, Londres, Inglaterra) y las condiciones de reacción establecidas por el fabricante.

### Consideraciones éticas

Se presentó una carta de información para resolver preguntas, y los pacientes y familiares que voluntariamente decidieron participar firmaron el consentimiento informado. El consentimiento familiar fue firmado por el paciente del caso índice autorizando al grupo de investigación ponerse en contacto con los parientes, indagar sobre su historia familiar e invitarlos a participar en el estudio.

Un consentimiento informado también fue firmado por los familiares del caso índice (sanos o afectados) que aceptaron participar libremente en el estudio.

Las entrevistas, protocolos y la propuesta de investigación se sometieron al Comité de Bioética de la Universidad del Tolima y cumplían a cabalidad con las normas nacionales e internacionales para la protección de la identidad.

## Resultados

Se seleccionaron por conveniencia 78 personas incluyendo los casos índice de las seis familias portadoras de la variante patogénica *BRCA1* 3450del4; 30 de ellas fueron positivas y sus análisis se repitieron y validaron en un centro de diagnóstico del exterior; los resultados se entregaron al médico tratante para iniciar el trámite de asesoría genética. De las 30 personas positivas para la variante patogénica, nueve habían desarrollado cáncer y 21 no presentaban ninguna neoplasia. Entre las 48 personas negativas para la mutación, dos tenían cáncer de mama y, uno, cáncer de próstata; a las otras 45 no se les había diagnosticado cáncer. Los resultados por familia se resumen en el cuadro 1.

**Cuadro 1:** Presencia o ausencia de la mutación *BRCA1* 3450del4 en seis familias del Tolima y del Huila

Código	Pacientes muestreados	Positivos para BRCA1 3450del4	Positivos afectados con cáncer	Positivos sin cáncer	Negativos afectados con cáncer	Negativos sin cáncer
Familia 1	13	3	1 CM y 1 CO	1	0	10
Familia 2	6	3	1 CM	2	0	3
Familia 3	11	5	1 CM	4	1 CM	5
Familia 4	18	9	1 CM y 1 CP	7	0	9
Familia 5	22	7	1 CM, 1 CM-CO	5	1 CM y 1 CP	13
Familia 6	8	3	1 CM	2	0	5
Total	78	30	9	21	3	45

CM: cáncer de mama; CO: cáncer de ovario; CP: cáncer de próstata; CM-CO: cáncer de mama y de ovario

**Figura 1:**
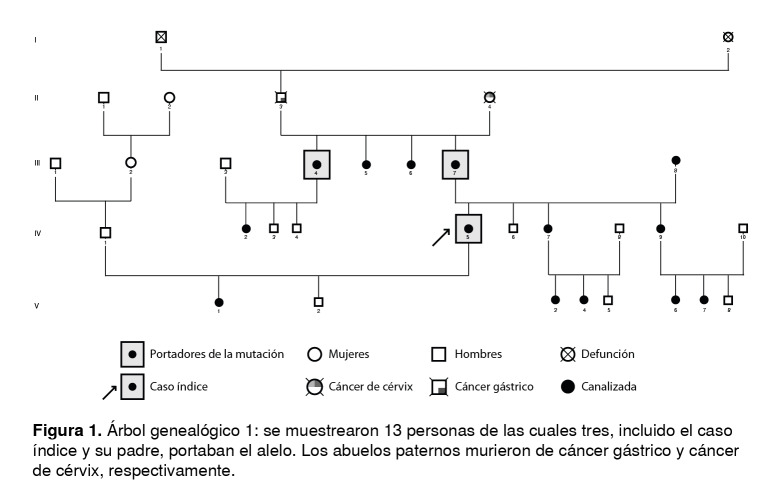
Árbol genealógico 1: se muestrearon 13 personas de las cuales tres, incluido el caso índice y su padre, portaban el alelo. Los abuelos paternos murieron de cáncer gástrico y cáncer de cérvix, respectivamente.

Las figuras 1 a 6 corresponden a los árboles genealógicos de los seis casos índice estudiados e incluyen la información molecular directa únicamente de las personas seleccionadas que voluntariamente aceptaron participar en el estudio y del caso índice con la variante patogénica *BRCA1* 3450del4; esta información se complementó con los datos indirectos derivados de las entrevistas anexas a cada una de las muestras, especialmente en cuanto a los diferentes tipos de cáncer.

**Figura 2:**
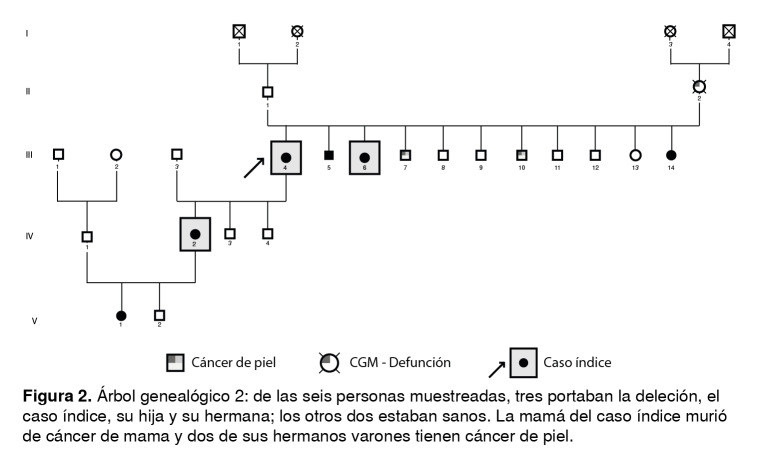
Árbol genealógico 2: de las seis personas muestreadas, tres portaban la deleción, el caso índice, su hija y su hermana; los otros dos estaban sanos. La mamá del caso índice murió de cáncer de mama y dos de sus hermanos varones tienen cáncer de piel.

**Figura 3:**
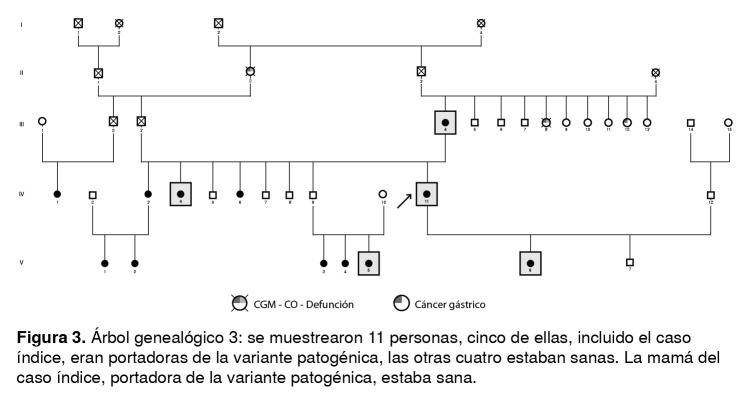
Árbol genealógico 3: se muestrearon 11 personas, cinco de ellas, incluido el caso índice, eran portadoras de la variante patogénica, las otras cuatro estaban sanas. La mamá del caso índice, portadora de la variante patogénica, estaba sana.

**Figura 4:**
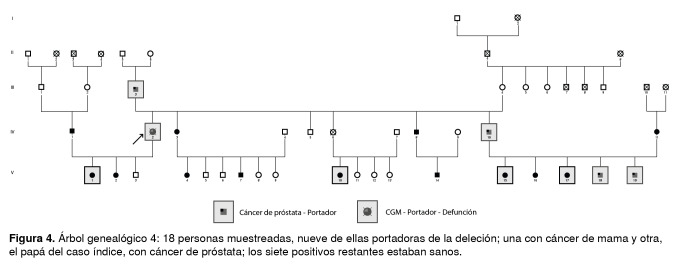
Árbol genealógico 4: 18 personas muestreadas, nueve de ellas portadoras de la deleción; una con cáncer de mama y otra, el papá del caso índice, con cáncer de próstata; los siete positivos restantes estaban sanos.

**Figura 5:**
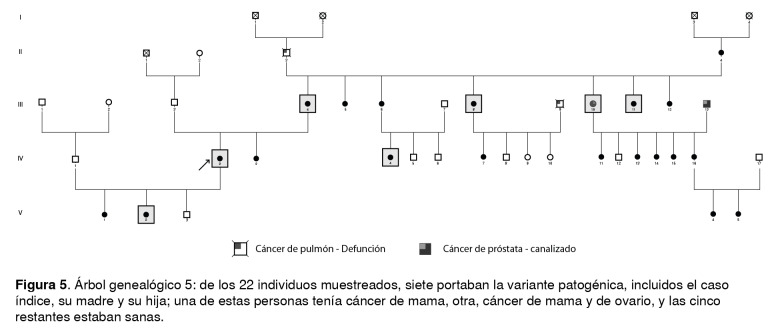
Árbol genealógico 5: de los 22 individuos muestreados, siete portaban la variante patogénica, incluidos el caso índice, su madre y su hija; una de estas personas tenía cáncer de mama, otra, cáncer de mama y de ovario, y las cinco restantes estaban sanas.

**Figura 6:**
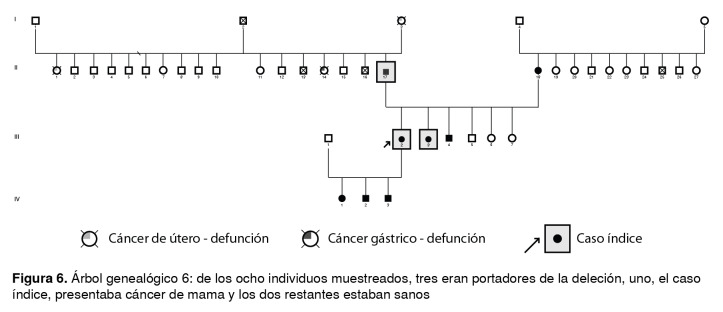
Árbol genealógico 6: de los ocho individuos muestreados, tres eran portadores de la deleción, uno, el caso índice, presentaba cáncer de mama y los dos restantes estaban sanos

**Figura 7:**
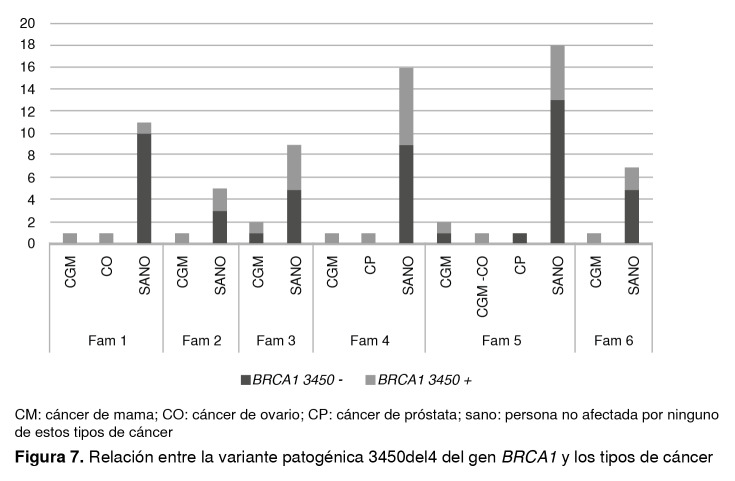
Relación entre la variante patogénica 3450del4 del gen *BRCA1* y los tipos de cáncer

CM: cáncer de mama; CO: cáncer de ovario; CP: cáncer de próstata; sano: persona no afectada por ninguno de estos tipos de cáncer

En la figura 7 se presenta la asociación del cáncer con la presencia de la variante patogénica *BRCA1* 3450del4 en cada una de las seis familias, y se evidencia que los tipos más comunes de cáncer en estas familias eran el cáncer de mama y el cáncer de ovario.

**Figura 8:**
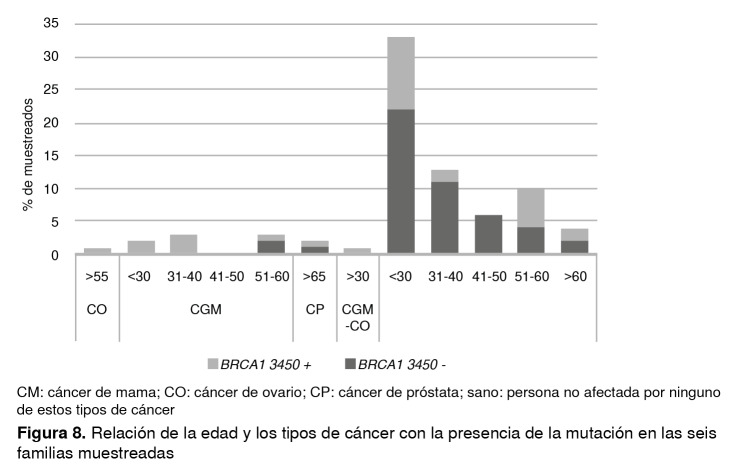
Relación de la edad y los tipos de cáncer con la presencia de la mutación en las seis familias muestreadas

CM: cáncer de mama; CO: cáncer de ovario; CP: cáncer de próstata; sano: persona no afectada por ninguno de estos tipos de cáncer

Por otra parte, los datos revelaron que el 70 % de los individuos seleccionados que presentaban la mutación no tenía cáncer en el momento del análisis de los datos; por esta razón, se estudió la distribución de los tipos de cáncer en relación con la edad (figura 8) y se evidenció que el 52 % de los 21 individuos positivos para la mutación que no habían desarrollado ningún tipo de neoplasia eran menores de 30 años; además, cinco de esas 21 personas positivas eran hombres.

## Discusión

Entre los factores involucrados en el desarrollo del cáncer de mama, se cuentan los genéticos y los ambientales ^18^. Los primeros incluyen la historia familiar de cáncer y la predisposición debida a la presencia de genes supresores de tumores, como el *BRCA1* y el *BRCA2,* ampliamente conocidos ^19^^,^^20^ por el riesgo que entrañan para desarrollar este cáncer y el de ovario. Las mutaciones de estos genes se han asociado con 5 a 10 % de todos los casos con agregación familiar y, de este porcentaje, aproximadamente, el 30 % está conformado por los casos de inicio de la enfermedad a edad temprana ^21^^,^^22^.

Los datos derivados de este estudio en seis familias del Tolima y del Huila confirmaron la asociación de la mutación deletérea 3450del4 del gen *BRCA1* con el cáncer de mama con agregación familiar, lo que coincide con: los reportes de Mahfoudh, *et al.*^12^, quienes la señalan como una de las cuatro encontradas en un estudio de 16 familias en Oriente medio y África del norte; los de Durocher, *et al.*^23^, quienes la encontraron en una de las 23 familias con cáncer de mama y cáncer de ovario que estudiaron; los de Torres, *et al.*^24^, que la encontraron en cinco de las 44 familias que estudiaron y la reportaron como una de las mutaciones fundadoras en Colombia de posible origen europeo y que explica buena parte del cáncer de mama con agregación familiar; estos autores afirman que en la base de datos del *Breast Cancer Information Core* (BIC) se reportan 21 familias europeas con la variante y una sola americana.

En un estudio de Briceño, *et al.*^25^ de 853 pacientes con cáncer de mama se reportaron 13 casos provenientes de Bogotá con la mutación *BRCA1* 3450del4 y dos del departamento del Tolima con las mutaciones C5214T y 5221 del T en el gen *BRCA1;* Hernández, *et al.*^7^^,^^15^, por su parte, identificaron en 244 pacientes con cáncer de mama en una zona de Medellín tres mutaciones deletéreas de las cuales una es la que nos ocupa. En otros estudios, entre ellos uno realizado en Chile en 64 familias con cáncer de mama y cáncer de ovario, se identificaron en siete de las familias analizadas seis variantes patogénicas del gen *BRCA1,* entre estas, Ia3450del4 ^15^^,^^26^; por último, en el norte de Brasil, a partir de datos de 106 pacientes con síndrome hereditario de cáncer de mama y ovario, se reportó la variante patogénica en cuatro de los individuos analizados ^15^^,^^27^.

Las mutaciones del *BRCA1* no solo están asociadas con el riesgo de cáncer de mama y cáncer de ovario, sino que presentan una fuerte asociación con el riesgo de cáncer de páncreas y de próstata en los ancestros fundadores, por ejemplo, los judíos ashkenazí, los islandeses y los fineses ^28^.

En las investigaciones realizadas por el Grupo de Investigación Citogenética, Filogenia y Evolución de Poblaciones, se ha detectado la mutación *BRCA1* 3450del4 en 28 casos de cáncer de mama con agregación familiar en el departamento del Tolima y del Huila, de los cuales se seleccionaron los seis casos índice de este estudio y en cuyas familias se muestrearon 78 individuos, 30 de ellos positivos para la deleción, nueve que habían desarrollado cáncer de mama, de ovario o de próstata, en tanto que los 21 restantes estaban sanos.

Frente a estos resultados cabe agregar que cinco de los 21 portadores sanos son hombres, lo que hace que el fenotipo tumoral sea más raro; además, ocho de las mujeres son menores de 30 años, lo que también llama la atención, pues, el cáncer se diagnostica con mayor frecuencia después de la menopausia, es decir, después de los 50 años ^29^. Además, para que la enfermedad se desarrolle, se requiere una segunda mutación (pérdida de heterocigocidad), pues el fenotipo que controla el genotipo heterocigoto es, en este caso particular, el resultado de una codominancia que permite que el alelo funcional produzca la proteína supresora normal de tumores, mientras el producto del gen mutado es una proteína truncada no funcional. Cabe resaltar que los individuos afectados por la pérdida de heterocigosis podrían presentar una penetrancia incompleta y una expresividad variable, pues, aparte de los factores genéticos, también los ambientales influyen de manera significativa en el desarrollo de este cáncer ^30^^,^^31^.

En este sentido, Caldés ^32^ postula que, aunque se herede un alelo mutado en el gen *BRCA1,* que teóricamente eleva de forma considerable el riesgo de desarrollar cáncer de mama y de ovario, existe una variabilidad genética y ambiental entre individuos que, en determinadas circunstancias, puede llevar a la reducción de la penetrancia del gen, es decir, el porcentaje de personas con el genotipo mutado que finalmente desarrolla la enfermedad.

Una de las posibles causas de la mutación *BRCA1* 3450del4 en los 28 casos con agregación familiar estudiados en el departamento del Tolima y del Huila por el Grupo de Investigación Citogenética, Filogenia y Evolución de Poblaciones, es el efecto fundador previamente reportado por Torres, *et al.,* entre otros ^13^^,^^24^. Asimismo, es importante tener en cuenta que, en las localidades muestreadas, especialmente en las huilenses, se observan cruces endogámicos frecuentes. Por último, los resultados del análisis de las seis familias extendidas, aunados a la mutación en los casos índice de las otras 22, confirman que la deleción estudiada es un factor importante de predisposición genética germinal para el desarrollo del cáncer de mama en la muestra regional.

Se recomienda seguir haciendo estudios en familias con historia de cáncer de mama para identificar esta deleción y continuar rastreando su posible efecto fundador.
